# Abrogation of Rb Tumor Suppression Initiates GBM in Differentiated Astrocytes by Driving a Progenitor Cell Program

**DOI:** 10.3389/fonc.2022.904479

**Published:** 2022-06-24

**Authors:** Amit S. Adhikari, Teresa Sullivan, Rhishikesh Bargaje, Lucy Lu, T Norene O’Sullivan, Yurong Song, Terry Van Dyke

**Affiliations:** ^1^ Mouse Cancer Genetics Program, Center for Cancer Research, National Cancer Institute, National Institutes of Health, Frederick, MD, United States; ^2^ Institute for Systems Biology, Seattle, WA, United States

**Keywords:** glioblastoma/astrocytoma, retinoblastoma tumor suppression, progenitors, cancer initiating cells, KRAS, cancer initiation

## Abstract

Glioblastoma (GBM) remains lethal with no effective treatments. Despite the comprehensive identification of commonly perturbed molecular pathways, little is known about the disease’s etiology, particularly in early stages. Several studies indicate that GBM is initiated in neural progenitor and/or stem cells. Here, we report that differentiated astrocytes are susceptible to GBM development when initiated by perturbation of the RB pathway, which induces a progenitor phenotype. *In vitro* and *in vivo* inactivation of Rb tumor suppression (TS) induces cortical astrocytes to proliferate rapidly, express progenitor markers, repress differentiation markers, and form self-renewing neurospheres that are susceptible to multi-lineage differentiation. This phenotype is sufficient to cause grade II astrocytomas which stochastically progress to GBM. Together with previous findings, these results demonstrate that cell susceptibility to GBM depends on the initiating driver.

## Introduction

High grade astrocytomas (HGAs), are the most common malignant brain tumors. Despite intense research and numerous clinical trials, overall survival time for patients has remained unchanged for 50 years, with diffuse astrocytoma (A2, World Health Organization (WHO); grade II) at 8 years, anaplastic astrocytoma astrocytoma (AA, WHO grade III) at 3-5 years), and glioblastoma (GBM; WHO grade IV) at 15 months (1). A TCGA study of 1,122 patients, identifying genomic alterations in GBM, indicates that a majority of tumors harbored aberrations in each of these signaling networks (the Rb network in 79%, RTK/Kras/PI3K/PTEN network in 90%, p53 network in 86% and all three networks in 74% of GBMs analyzed) (2). Genetically engineered mice (GEM) have proved essential in establishing cause/effect relationships for many commonly occurring GBM molecular perturbations (3–5). Moreover, because staged patient samples are not accessible, our limited understanding of disease etiology has been deciphered in GEMs. Yet, defining the mechanisms of GBM initiation and stochastic progression is especially challenging, since most studies depend on multiple simultaneous engineered genetic lesions to elicit disease. Furthermore, early GEM-GBM stages have rarely been characterized. Nonetheless, by targeting GBM-causal allele combinations to specific cell types, several studies have identified potential tumor initiating cells (TICs). Most reports indicate that neural stem cells (NSCs) and/or progenitor cells are the target for GBM initiation. For example, co-deletion of *Trp53*, *NF1*, and *PTEN* by intracranial adenovirus-Cre injection elicited GBM when targeted to the adult subventricular zone (SVZ; rich in adult stem and progenitor cells), but not to the differentiated cells of the cortex (6). Similar co-deletion of *Trp53* and *PTEN* in the SVZ also promoted GBM-like histology (7). Lineage tracing studies subsequent to *Trp53* and *NF1* deletion implicated oligodendrocyte precursor cells as a GBM target (8); however, since mutations were induced developmentally with constitutive germline Cre drivers, this result requires verification in adult mice.

In contrast, we showed that GBM is efficiently induced in the adult frontal cortex in response to lentiviral-Cre-induced Rb tumor suppression (Rb-TS) inhibition, K-Ras activation, and PTEN deletion (9). Additionally, approximately 50% of GEM-GBMs stochastically develop p53 missense mutations identical to those observed in human GBMs. Furthermore, adult astrocyte-specific induction of drivers singly, and in combination, showed that only Rb-TS inhibition could initiate disease and was sufficient to yield diffuse grade II disease. Additional activation of K-Ras^G12D^ was required for progression to high grade tumors, predominantly grade III (AA). Further engineered or stochastic deletion of *PTEN* drove progression from AA to GBM. Mutant p53 appeared in foci subsequent to K-Ras activation and prior to AA mass formation and thus has a role in the transition from low to high grade disease (9).

Both our study and that of Alcantara Llaguno et al. utilized intracranial Cre-mediated induction to perturb the K-Ras/NFI, p53, and PTEN pathways. However, our evaluation, which yielded GBM upon driver induction in the cortex, included the inhibition of Rb-TS. Hence, we reasoned that Rb-TS inhibition could be responsible for rendering differentiated astrocytes susceptible to GBM development. Since this activity could initiate astrocytoma in differentiated astrocytes (9), while the other drivers could not, a requirement of initiation was to render astrocytes susceptible to K-Ras-mediated progression. Given that Rb is known to drive withdrawal from the cell cycle upon differentiation of many cell types (10–14), we hypothesize that abrogation of Rb and compensatory proteins p107 and p130 (collectively responsible for tumor suppression in murine astrocytes) initiates GBM in differentiated astrocytes by inducing a progenitor phenotype, thus generating a highly proliferative cell population with sensitivity to drivers of progression. Indeed, the studies herein show that Rb-TS inactivation induces the transition of mature astrocytes to multipotent progenitor-like cells, thus initiating disease which stochastically progresses to yield high grade astrocytomas.

## Material and Methods

### Genetically Engineered Mice and Systemic Induction

*TgGZT_121_* conditional transgenic mice were maintained by crossing to BDF1/J mice (15) *KRAS^G12D^* conditional knock-in mice (16), and *hGFAP-CreER^T2^* (17) mice to C57Bl/6J mice. Germline genotyping was performed as previously described (15, 16). Genetic events were induced by injecting freshly prepared 4-OH-tamoxifen (4OHT, Sigma, St. Louis, MO) intraperitoneally into 2 or 6 month old adult mice with 1 mg/mouse/day for 5 consecutive days. 4OH tamoxifen is dissolved in 90% filtered sunflower seed oil (Sigma Aldrich)/10% EtOH (vehicle) with final concentration at 1mg/100ul and sonicated by a dismembrator to create an emulsion. Methods described herein have been approved by the Institutional Animal Care and Use Committees at the National Cancer Institute, Frederick, MD.

### Focal Induction by Stereotaxic Intracranial Injection

Stereotaxic injection was as described previously (18). Mice were anesthetized with isoflurane and placed into a stereotaxic frame (KOPF). One microliter of self-deleting recombinant lentivirus (~10^9^ particles per ml) (Salk Institute for Biological Studies Viral Vector Core) expressing Cre recombinase (pBOB-CAG-iCRE-SD, a kind gift from Dr. Katrin Zimmermann, University of Bonn). Coordinates used were: (A, L, D) = 1.5, 1.5, and 1.2 mm from the Bregma suture targeting the frontal cerebral cortex in the brain.

### Microarray Transcriptome Analysis

Microarray data were processed and analyzed with R/Bioconductor packages and Gaggle software platform (19). The quality of raw data was first checked from QC reports and one of the arrays was removed at this stage. Sample information for the arrays that were used for subsequent analysis is provided in **Table 1**.

**Table 1:** Information about samples used for the microarray analysis.

Genotype	Weeks post induction	Number of samples
TC+	2	4
TC+	4	4
TC+	8	3
T	8	4

Sample information regarding the mouse genotype and time post induction of 4OHT along with the number of samples. TC+, TgGZT_121_, GFAP-CreER^T2^ mice after being treated with 1mg/mouse/day 4OHT for 5 consecutive days; T, TgGZT_121_ mice without Cre.

In-house python scripts were used to perform background correction of the data. The data was then log_2_ transformed and quantile normalized. All raw data are publicly available (GSE26069). We used this data to identify differentially expressed genes using two independent methods: i) time series dependent: BETR, and ii) comparison of each time point to control with one-way ANOVA.

### BETR

The Bayesian Estimation of Temporal Regulation (BETR) algorithm has been shown to increase the power of detecting the subtle changes in expression of genes in a time-series data (20). We therefore, used BETR (21) (available through MeV) to identify 2,164 probes significantly (uncorrected p < 0.01) expressed across the time series from 2 to 8 weeks post induction. Furthermore, we compressed the expression for multiple probes representing same transcript and filtered probes with standard deviation greater than 1, to generate list of 1,440 transcripts. We find 638 transcripts to be significantly differentially expressed in the time series as well as fold change >= 2 in at least one of the time point comparisons.

### T-Tests

We used MeV (21) to perform Welch’s t-tests to identify the differentially expressed genes for each time point as compared to control samples. We estimated the p-values based on 1,000 permutations of genes between each group. We used a cut off of p > 0.01 for standard Bonferroni corrected p-values. Finally, we compressed the data for multiple probes for the same transcripts, removed the transcripts with standard deviation greater than 1 across samples, and applied an additional cut off of fold-change = 2 to derive lists of the differentially expressed genes for all comparisons.

#### GSEA

We used the normalized data on desktop version of GSEA v2.0.13 available from the broad institute website (http://www.broadinstitute.org/gsea/downloads.jsp). We converted the data in requisite format to be used as input for the analysis. Similar to differential gene expression, we performed GSEA as described in (22); in mainly two ways: i) continuous phenotype: time series and ii) categorical phenotype: individual time point comparisons. The following gene sets were used for the analysis: c2.all.v4.0, c3.tft.v4.0, c4.all.v4.0, c5.bp.v4.0, c5.mf.v4.0, c6.all.v4.0 and c7.all.v4.0; excluding gene sets with less than 12 or more than 500 genes. The expression data was collapsed based on gene symbols and p-values were generated based on 1,000 permutations in gene sets. The metric for ranking the genes was t-tests, with appropriate multiple hypothesis testing.

### Statistical Analysis

Student’s t-test was performed for mean comparisons of continuous variables. All statistical tests were two-sided and p < 0.05 was considered significant unless otherwise stated.

Additional material and methods are noted in the supplementary section.

## Results

### Astrocyte-Specific Rb-TS Inactivation Induces a Progenitor State Signature

Inhibition of the Rb tumor suppressor (Rb-TS) function in most murine cell types requires inhibition of its compensatory family members, p107, and p130 (23–25). We utilized T_121_, the N-terminal 121 amino acid fragment of SV40 large T antigen which dominantly inactivates all three Rb family members. The hGFAP promoter drives the conditional T_121_ transgene in astrocytes and CreER^T2^ ensures that this genetic alteration is inducible by estrogen analog 4OHT. TgGZT_121_; GFAP-CreER^T2^ mice (hence forth referred as TC) after being treated with 4OHT (TC+) induced T_121_, leading to Rb-TS inactivation. This has previously been shown to lead to hyperproliferation of cells with Grade II astrocytoma (9). To determine whether the transcriptional changes induced by Rb-TS inactivation were consistent with our hypothesis that astrocytes transition to a progenitor-like state, we analyzed cortical tissue from TC+ mice at 2, 4, and 8 weeks post-induction (p.i) (4 mice per group). These tissues were compared to the normal cortex from 8 week old TgGZT_121_ mice without Cre (T). We used Custom Agilent SurePrint microarrays and probes for 24,510 unique Entrez Gene ids. We find 638 transcripts significantly differentially expressed in the time series with a fold change ≥ 2 in at least one of the time-point comparisons.

Significantly induced gene sets through Gene Set Enrichment Analysis (GSEA) in TC+ cortex indicate activation of cell cycle activity, cell plasticity, and progenitor phenotype (**Table 2**). As expected, based on perturbation of Rb function, Rb and E2F activities were represented in the induced cell cycle gene sets. Consistent with the induction of progenitor phenotype, numerous stem/ES cell gene sets were enriched. Increased expression of stem/progenitor genes Ezh2, Notch1, Notch2, Gli2, Bcl2L12, Nanog, N-Myc, and ID4 was confirmed by qPCR RNA analysis (**Figures S1A, B**). Interestingly, even at the early stage in astrocytoma genesis, TC+ cortex showed an increase in gene sets comprising GBM neural/proneural subtype, along with additional astrocytoma related gene sets (**Table 2**).

**Table 2:** GSEA data showing gene data sets upon Rb-TS inactivation.

Gene sets	Size	NES	NOM p value	FDR q value
**Cell Cycle**				
CHANG_CYCLING_GENES (148)	131	-4.11	0	0
WHITFIELD_CELL_CYCLE_LITERATURE (44)	43	-3.48	0	0
EGUCHI_CELL_CYCLE_RB1_TARGETS (23)	23	-3.46	0	0
BENPORATH_PROLIFERATION (147)	136	-3.13	0	0
**Rb Targets**				
KONG_E2F3_TARGETS (97)	90	-3.82	0	0
ISHIDA_E2F_TARGETS (53)	51	-3.70	0	0
KAMMINGA_EZH2_TARGETS (41)*^1^	41	-3.51	0	0
REN_BOUND_BY_E2F	57	-3.36	0	0
CHICAS_RB1_TARGETS_GROWING (241)	187	-2.67	0	0
**Plasticity and Progenitor phenotype**				
RHODES_UNDIFFERENTIATED_CANCER (69)*^2^	65	-3.23	0	0
WONG_EMBRYONIC_STEM_CELL_CORE (335)	321	-2.83	0	0
BENPORATH_ES_2 (40)	35	-2.25	0	6E-04
BENPORATH_ES_1 (379)	343	-2.20	0	8E-04
BENPORATH_OCT4_TARGETS (290)	265	-1.27	0.02	2E-01
**Astrocytoma and Glioblastoma**				
ZHENG_GLIOBLASTOMA_PLASTICITY_UP (250)*^3^	232	-2.83	0	0
JOHANSSON_BRAIN_CANCER_EARLY_VS_LATE_DN (45)	45	-2.18	0	1E-03
VERHAAK_GLIOBLASTOMA_NEURAL (210)	194	-2.08	0	2E-03
COLIN_PILOCYTIC_ASTROCYTOMA_VS_GLIOBLASTOMA_DN (28)	28	-2.06	0	3E-03
MUELLER_METHYLATED_IN_GLIOBLASTOMA (40)	34	-1.77	0.01	2E-02
ZHENG_GLIOBLASTOMA_PLASTICITY_DN (58)*^4^	57	-1.37	0.04	1E-01
VERHAAK_GLIOBLASTOMA_PRONEURAL (210)	219	-1.22	0.05	2E-01

*1- Included in Cell Cycle genesets. *2- Included in Astrocytoma and Glioblastoma genesets. *3- Included in Plasticity and Stemness genesets. *4- Included in Plasticity and Sternness genesets.

### Rb-TS Abrogation Transitions Mature Astrocytes to Progenitor Phenotype *In Vitro*

**Figure 1:**
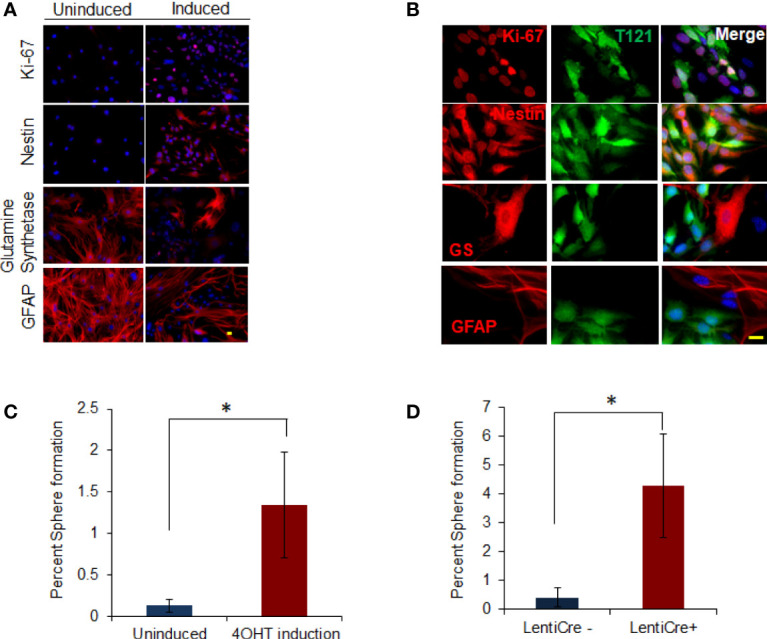
*In vitro* dedifferentiation of astrocytes. **(A)** Images showing uninduced and induced astrocytes after 4OHT addition stained with Ki-67 and nestin, glutamine synthetase and GFAP (in Red) using immunofluorescence. Nuclei counterstained with DAPI (blue). Scale bar indicates 20 micron. **(B)** Images showing astrocytes induced with 4OHT and co-stained with T_121_(Green) and Ki-67, nestin, glutamine synthetase (GS) and GFAP (Red) respectively using immunofluorescence. Nuclei counterstained with DAPI (blue). Scale bar indicates 20 micron. **(C)** Histogram showing percent sphere formation from *in-vitro* progenitor-like transition of T astrocytes. Blue bar indicates percent sphere formation from uninduced astrocytes and red bar shows percent sphere formation upon 4OHT induction. N-5 mice and 4000 astrocytes per sample where analyzed. * indicates p value < 0.05%. **(D)** Histogram showing percent sphere formation from *in-vitro* progenitor-like transition of triple knockout Rb lox/lox, p107-/-, p130 lox/lox astrocytes. Cre recombinase is introduced in the cells by lenti virus infection. Blue bar indicates percent sphere formation from uninduced astrocytes and red bar shows percent sphere formation upon lenti-cre induction. N-6 mice and 4000 astrocytes per sample where analyzed. * indicates p value < 0.05%.

Since the progenitor/stem cell signatures were induced in TC+ cortex, we investigated whether Rb-TS inactivation could functionally induce a progenitor-like phenotype in isolated mature astrocytes. Astrocytes were isolated from postnatal (PN) 5-7 days old T mice (uninduced TgGZT_121_; GFAP-CreER^T2^ astrocytes referred to as TA). TA cells were cultured until they ceased cell division and displayed the morphology of terminally differentiated astrocytes (generally after 3-4 passages; **Figure S2**). At this stage, 85% of the cells stained positive for the astrocyte differentiation markers- Glial Fibrillary Acidic Protein (GFAP) and Glutamine Synthetase (GS). Only 2% of the cells showed S100β positivity indicating enrichment of a subtype of astrocytes. Based on marker staining, a small percentage of additional cell types were detected (oligodendrocytes 1.5% and neurons 12.7%) with the absence of MAP2-positive mature neurons (**Figure S2**). Using Ki-67 immunocytochemistry, it was observed that upon T_121_ induction with 4OHT, astrocytes (induced TgGZT_121_; GFAP-CreER^T2^ astrocytes referred to as TA+) re-entered the cell cycle as early as 4 days post induction (p.i) (**Figures 1A, B**). Proliferating cells also expressed neural progenitor marker nestin (26, 27), (**Figures 1A, B**). Dual staining with T_121_ confirmed the co-expression of Ki-67 and nestin with T_121_(**Figure 1B**), indicating that the reentry of cell cycle and expression of progenitor marker were a T_121_ specific phenomenon. T_121_ showed nuclear and cytoplasmic distribution with variable intensities, whereas Ki-67 and nestin localized to the nucleus and cytoplasm respectively (**Figure 1B**). In addition to the expression of proliferation and progenitor markers, immunocytochemistry showed that the expression of differentiated astrocyte markers GS and GFAP (26–28), reduced upon T_121_ induction. While both these markers were prominent in uninduced cultures, only few cells expressed GS and GFAP after induction and this reduction was specific to T_121_ expressing cells (**Figures 1A, B**). Cell morphology, being consistent with the expression of progenitor markers and suppression of differentiation markers, also transformed from large, flat cells with several astral processes to small, spindle shaped cells (**Figure 1A** GS, GFAP). These results indicated that T_121_ (Rb-TS loss) drives mature astrocytes to express a stem/progenitor-like phenotype.

To determine whether marker status was indicative of functional transitioning of astrocytes into progenitor cells, we tested them for neurosphere forming ability. Neurophere assay on Rb-TS inactivated TA+ cells *in vitro* produced neurospheres at a tenfold greater efficiency than uninduced cells (1.34% vs 0.13%, respectively) (**Figure 1C**). The few neurospheres produced from uninduced cells expressed T_121_ indicating residual CreER^T2^ activity in the absence of 4OHT. The specificity of T_121_ (N terminal fragment of SV40 large T antigen) to inhibit Rb family members, has been shown by our group previously (29) but the possibility of an independent role still remains. We performed *in vitro* progenitor transition assay using mature astrocytes isolated from Rb3 mouse (Rb and p130 conditional knockout and p107 conventional knockout mouse) to rule out this possibility. Neurosphere assay (**Figure 1D**) in induced Rb3 astrocytes, showed sphere formation as was observed in TA+ astrocytes. Formation of spheres was significantly higher in Rb3 compared to TA+ astrocytes (4.3% and 1.3% respectively). Marker profiles observed in Rb3 astrocytes were identical to that of TA+ astrocytes (data not shown). These results ruled out any independent role for T_121_, and confirmed that *in vitro* Rb-TS inactivation induced progenitor-like transition of astrocytes.

In order to investigate the multiple differentiation ability of the transitioned progenitor cells into neural lineages, we subjected these cells to differentiation conditions. Upon induced differentiation, the T mice SVZ sphere cells (control cells) differentiated into astrocytes, neurons, and oligodendrocytes (**Figure 4B**). At day zero, the control and TC+ sphere cells showed progenitor cell morphology with the absence of differentiation markers - S100β(astrocyte), MAP2 (neuron), and GalC (oligodendrocyte). GFAP was expressed in cells showing progenitor-like morphology, due to its known expression in adult stem cells (30). TC and control cells also expressed Tuj 1 (early neural marker, 10%) and NG2 (early oligodendrocytic marker, 10%) (**Figure 4B** day 0). Upon differentiation, TC+ cells showed increased expression of Tuj 1 (50%) and NG2 (50%), in addition to the presence of mature differentiation markers S100β, GFAP (astrocytic lineage), MAP2 (neural lineage) and GalC (oligodendritic lineage). This showed that the TC+ sphere cells possessed multilineage differentiation ability under appropriate culturing conditions.

### *In Vivo* Cortical Astrocytes Transitions Into Progenitors Upon Abrogation of Rb-TS

**Figure 2:**
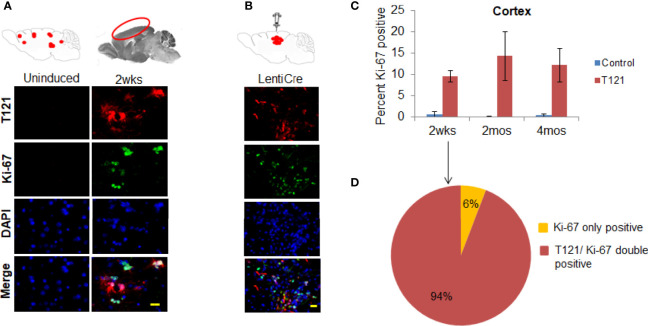
T_121_ and Ki-67 staining and quantitative analysis in the cortex. **(A)** Immunofluorescence images of brain sagittal sections of 3 month old mouse induced with intraperitoneal injection of 4OHT for 2wks post induction and uninduced brain section as control. Cartoons at the top represent global induction events of T_121_ and the cortical area being visualized in the fluorescent images. Images of the cortical area have T_121_ in red, Ki-67 in green, merge and nuclei counterstained DAPI is blue. Scale bar indicates 20 micron. **(B)** Immunofluorescence images of brain sagittal sections of a 3 month old mouse with focal induction using lentiCre for 2wks post induction. Cartoons at the top represent focal induction events of T_121_ in the cortex. Images of the cortical area have T_121_ in red, Ki-67 in green, merge and nuclei counterstained DAPI is blue. Scale bar indicates 20 micron. **(C)** Quantitative analysis of Ki-67 positive cells in the cortex. Histogram of percent Ki-67 positive cells in the cortex from control and T_121_ induced mice at different time points -2wks, 2mos and 4mos post induction. Blue bars- Control mice, Red bars- T_121_ induced mice. N-5 mice and 3000 cells from 6 random fields/mouse. **(D)** Pie chart depicting the overlap between Ki-67 and T_121_ positive cells from a 2wks post T_121_ induced cortex. 1200 Ki-67 positive cells from 50 different fields and 6 mice were visualized for an overlap with T_121_ staining.

**Figure 3:**
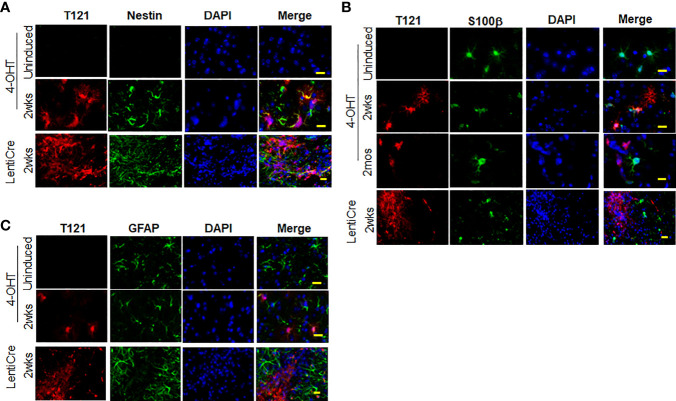
Expression of nestin, S100β and GFAP upon Rb-TS inactivation in GEMM. **(A)** Immunofluorescence of sagittal sections of brain denoting cortical area in uninduced, T_121_ globally (IP 4OHT injection) induced 2wks post induction and focally induced using lenti virus expressing Cre recombinase (lenti-Cre) 2wks post induction. T_121_-red, nestin-green, merge and DAPI (nuclei) in blue. Scale bar indicates 20 micron. **(B)** Immunofluorescence of sagittal sections of brain denoting cortical area in uninduced, T_121_ globally (IP 4OHT injection) induced 2wks and 2mos post induction and focally (LentiCre) induced 2wks post induction. T_121_-red, S100β-green, merge and DAPI (nuclei) in blue. Scale bar indicates 20 micron. **(C)** Immunofluorescence of sagittal sections of brain denoting cortical area in uninduced, T_121_ globally (4OHT) induced 2 wks post induction and focally (LentiCre) induced 2wks post induction. T_121_-red, GFAP-green, merge and DAPI (nuclei) in blue. Scale bar indicates 20 micron.

To confirm the transition of mature astrocytes to progenitor cells *in vivo*, we analyzed mature astrocytes in globally and focally induced genetically engineered mice models (GEMMs). Immunohistochemistry (IHC) results showed that upon global inactivation of Rb-TS, both T_121_ and Ki-67 co-expressed at 2 weeks p.i., while they were totally absent in uninduced cortex (**Figure 2A**). A similar result was obtained upon focal induction (**Figure 2B**). Quantitative evaluation of IHC staining confirms the increase in Ki-67 positive cells p.i (**Figure 2C**). Ninety four percent of Ki-67 positive cells co-expressed T_121_, confirming that proliferation is a T_121_ specific event (**Figure 2D**). As observed in the case of Ki-67, upon global and focal inactivation of Rb-TS, nestin and T_121_ markers were also co-expressed, which is indicative of a progenitor-like phenotype (**Figures 3A, B**). In the presence of T_121_, we observed that astrocyte differentiation markers - S100β and GFAP, demonstrated either partial or complete loss (**Figures 3B, C**). These results further corroborated our conclusion that even under *in vivo* conditions, mature astrocytes transition into progenitor-like cells upon inhibition of Rb family members. GFAP promoter driving T_121_ was also found to be active in adult stem cells leading to inactivation of Rb-TS in these cells (30). Thus, the observations using focal induction of Rb-TS inactivation was crucial to eliminate the involvement of adult stem cells in the observed progenitor-like phenotype.

**Figure 4:**
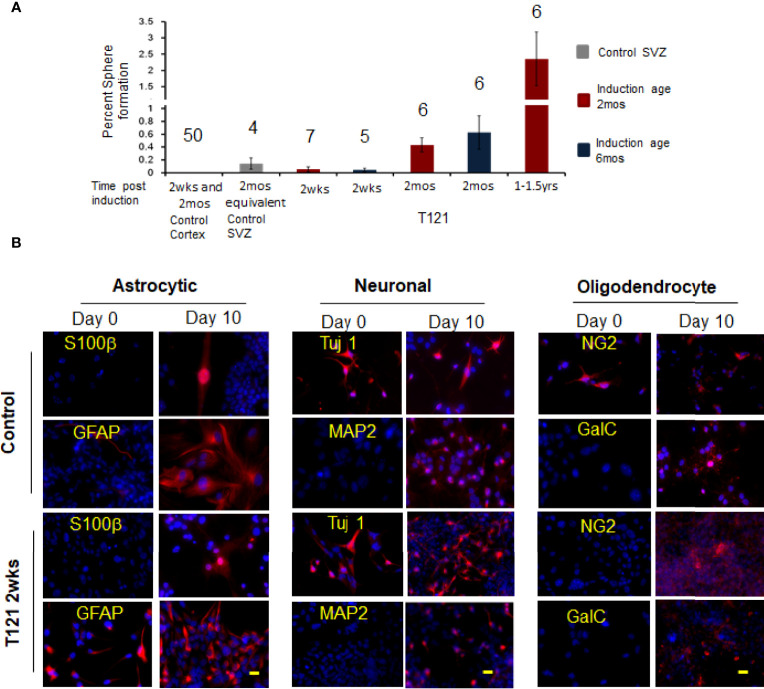
*In vivo* progenitor-like cells have the ability to form spheres and differentiate into multiple neuronal lineages. **(A)** The histogram shows percent sphere formation for cortical cells isolated from either control mice or 4OHT induced mice at different time points. The mice were either induced at the age of 2 mos. red bars or induced at the age of 6 mos. blue bars. The gray bar is sphere formation from SVZ cells which are control cells to evaluate the assay conditions. The time post induction is indicated below each bar. There were 4,000 cells used for each assay and the number above the bar indicates the number of mice evaluated for each time point. **(B)** Images showing presence of differentiation markers for the three neuronal lineages. Within each neuronal lineage, top panel shows differentiation of control neurosphere cells (adult stem cells isolated from 3mos old wildtype mouse). Bottom row is differentiation of neurosphere cells from T_121_ 2wks post induced cells. Left column shows neurosphere cells at day 0 and Right column differentiated cells. Cells were cultured in absence of growth factors and presence of serum for 10 days to differentiate into the three lineages. Astrocytes markers S100β (Red) and GFAP (Red) were used to indicate differentiation. For neuronal lineage Tuj1 (Red) and MAP2 (Red) and for oligodendrocytic lineage NG2 (Red) and GalC (Red). Nuclei counterstained with DAPI (blue). Scale bar indicates 20 micron.

When the *in vivo* progenitor population was challenged with neurosphere assay, the control wt cortical cells failed to generate neurospheres despite numerous attempts (N=50). SVZ cells, representing adult stem cells (control 4 month old mice) showed 0.14% sphere formation, confirming the viability of the assay (31, 32). Upon induction of T_121_, the cortical cells were able to generate spheres and the percentage of sphere formation increased with time (2 weeks 0.07%, 2 months 0.43%, and 1-1.5 year 2.4%) (**Figure 4A**, red bars). These spheres were successfully cultured at clonal density for more than four generations, confirming their self-renewal ability. Thus, we conclusively demonstrated that inactivation of Rb-TS and transitioned mature cortical astrocytes into progenitor-like cells under both *in vitro* and *in vivo* conditions.

Q-PCR analysis for validating the increased expression of stem/progenitor genes (Ezh2, Notch1, Notch2, Gli2, Bcl2L12, Nanog, N-Myc, and ID4) was observed in GSEA analysis and was done using neurosphere cultures. Using wildtype astrocytes (postnatal 5-7 day old mice) as control, the increased expression of the above mentioned genes in neurospheres from TC+ was confirmed indicating cell-autonomous expression of the stem/progenitor genes (**Figure S1B**).

### Transition to Progenitor Cells Is T_121_ and Mature Astrocyte- Specific Phenomenon

To eliminate the involvement of normal cortical progenitor cells (33) in the astrocyte-transition, we induced T_121_ in mice at 6 months instead of inducing at 2 months, considering the fact that the number of normal cortical progenitor cells reduce with age. We observed a similar percentage of spheres generated from cortical cells from mice induced at 6 months and those induced at 2 months (**Figure 4A**, blue bars, 2weeks and 2 months), eliminating the involvement of cortical progenitor cells in the transition of astrocytes to progenitor-like cells. The above experiments collectively confirmed that the normal adult stem or progenitor cells are not involved in the transition of adult astrocytes into progenitor cells.

Mice with abrogation of Rb-TS promoted grade II astrocytomas without any solid tumor mass within 6 months, but when layered with Kras activation mutant (KRAS^G12D^), developed grade III and grade IV high grade astrocytomas, with 4-6 months of latency (Mice with genotype KRAS^G12D^; GFAP*-*CreER^T2^ will be referred as R and TgGZT_121_; KRAS^G12D^; GFAP-CreER^T2^ as TR) (9). Since addition of Kras activation led to increase in the grades of astrocytoma, we investigated its role in transitioning of astrocytes to progenitor-like cells. Neurosphere assay using cortical cells from R mice failed to generate any spheres (**Figure S3**). Upon induction of TR mice, the cortical cells showed sphere formation similar to that of TC+ cells (TR 2 weeks - 0.07%, TR 2 months - 0.34%) (**Figure S3** blue bars). Thus, we conclude that Kras activation individually or along with Rb-TS inactivation does not play a role in astrocytes transitioning into progenitor like cells.

### Mice With Single Genetic Alteration of Rb-TS Inactivation Develop HGAs With a Long Latency

**Figure 5:**
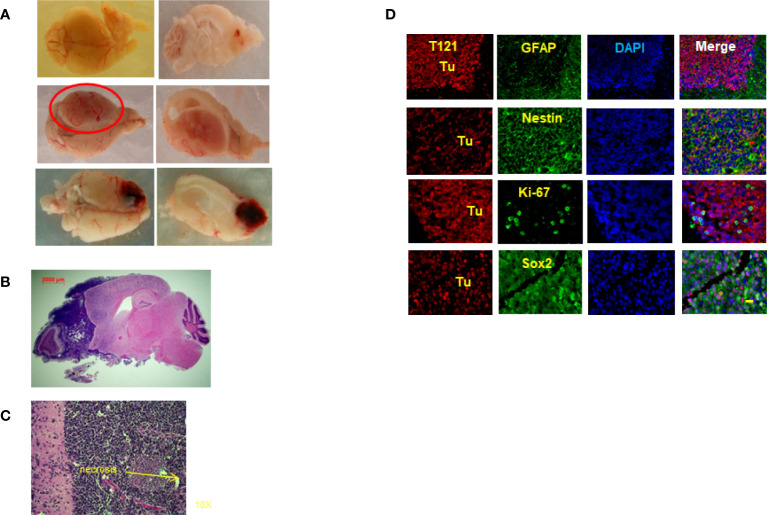
Rb-TS inactivated mice develop High Grade Astrocytomas. **(A)** Representative images showing High Grade Astrocytomas at different locations in the brain with whole brain (left side) and brain and tumor sagittally sectioned (right side). **(B)** Whole sagittal section of the brain, H&E stained showing the High Grade Astrocytoma. **(C)** Representative zoomed in image of H&E stained tumor image showing characteristic necrosis and pleomorphic nuclei in an High Grade Astrocytoma. **(D)** Immunofluorescence of sagittal sections of the brain with High Grade Astrocytoma T_121_ showing T_121_-red, GFAP, Nestin, Ki-67, Sox2-green, merge and DAPI (nuclei) in blue. Tu indicate tumor area. Scale bar indicates 20 micron.

To prove our hypothesis that Rb-TS inactivation transitions mature astrocytes into progenitors, generating potential cell(s) of origin for astrocytoma, we aged TC+ mice under observation for astrocytoma formation. Interestingly, after a latency period of 15-24 months, the TC+ mice developed HGAs with a penetrance of 68%. Most of the tumors developed in the frontal cortex of the brain with occasional inclusion of olfactory bulbs (**Figures 5A, B**); while few mice developed tumors in brain midcortex and spine regions (**Figure 5A**). Pathologically, the tumors showed HGA characteristics of pleomorphic and anaplastic nuclei, accompanied by necrosis in Grade IV astrocytomas (**Figure 5C**). Tumor IHC demonstrated the presence of T_121_ throughout the tumor, confirming their origin from Rb-TS inactivated cells. Further IHC staining of tumor for HGA markers including GFAP, Sox2, nestin, and Ki-67 confirmed the HGA status of the brain tumors (**Figure 5D**). Thus, mature astrocytes initiated by Rb inactivation alone can develop into astrocytoma with long latency.

## Discussion

Most of the previous reports on the origin of GBM have suggested adult stem cells (6, 7) and precursors (8) to be the cell/cells of origin. Here, we show that the target cell depends on the nature of the genetic event which arises. Using adult inducible mouse model for HGAs, we demonstrate that cortical astrocytes upon Rb-TS inactivation transition into progenitor-like cells, generate neurospheres, possess self-renewal ability, and differentiate into the three neuronal lineages. Transitioned cells were susceptible to additional genetic perturbations leading to generation of HGAs in Rb-TS inactivated mice.

### Role of Rb Network in Differentiation and Cancer

Perturbation of Rb function occurs in most sporadic solid malignancies. While Rb itself is inactivated in some cancer types, several of its upstream regulators, including p16INK4a, cyclin D1, and CDK4, are more commonly mutated resulting in compromised Rb function.

In the current study, global analysis of RNA from cortical cells showed a progenitor-like transition of astrocytes. Enrichment of gene sets for embryonic stem cells and GBM plasticity were of particular interest, as these not only support our conclusion of progenitor-like transition, but also provide evidence for the similarities between our GBM initiation model and the human disease. As expected, we observed enrichment of cell cycle and Rb downstream target gene sets due to the involvement of Rb family. The enrichment of the Verhaak GBM subtype, neural/proneural was, however, surprising. Further studies could help shed light on the origin of this subtype. Upon Rb-TS inactivation in mature astrocytes *in vitro*, cells changed their morphology, entered cell cycle, expressed progenitor marker nestin and reduced differentiation markers. These cells generated neurospheres which could be maintained for more than four generations at clonal densities indicating their self-renewal ability. These results indicate that the mature astrocytes reversed their differentiated status into a progenitor state under *in vitro* conditions. The pRb role in cell-cycle control at the G1-S transition (by E2F transcription factor inhibition and chromatin modulation) is well studied. Under normal circumstances, its primary role is in withdrawal from the cell cycle during differentiation (25) and loss of Rb and/or other pocket proteins lead to cell cycle re-entry of quiescent stem and post-mitotic differentiated cells (10–12). Astrocytes have been shown to transition to progenitor phenotype *in vitro* upon overexpression of Id4 in Ink4a/ARF^-/-^ astrocytes (34), Ezh2 (35), Nanog and AKT, cMyc in p53 null astrocytes (36, 37) and also TNF-α in normal astrocyte cultures (38). However, the link between dedifferentiation and tumorigenesis remains unexplored and also, in most cases, multiple genetic events are required to achieve dedifferentiation.

To investigate the progenitor like transition under *in vivo* conditions, we used the TR GBM model where Rb-TS is abrogated along with Kras activation (9). Systemic induction of CreER using 4OHT and cortex-specific induction (mature astrocytes specific) *via* Cre-lentivirus both developed HGAs within 6 months suggesting that HGAs can originate independently of the germinal areas in the brain. Our immunohistochemistry results from TC+ mice systemic and focally induced brains demonstrate cell cycle reentry and proliferation to be the important steps in the progenitor transition in post-mitotic cells (12). Expression of progenitor marker nestin and reduction of differentiation markers in TC+ cortical cells was specific to the Rb-TS inactivated cells and similar to *in vitro* observations. The transitioned cells formed neurospheres and percent neurosphere formation was directly correlated to the post induction period. It is important to note that the wildtype cortical cells failed to generate any spheres even after multiple attempts. Neurosphere cells showed self-renewal ability and multi-lineage differentiation into astrocytes, neurons, and oligodendrocytes— an important requirement to achieve the intratumoral heterogeneity and differentiation hierarchy observed in HGA.

### Mature Astrocytes as a Cell of Origin for Tumor-Initiating Cells and Glioblastoma

Cancer stem cells (CSCs) or TICs were identified in brain tumors by Singh et al. in 2004 (39) and since then have been shown to possess lethal characteristics of HGAs, namely chemo- and radio-therapy resistance, invasion, and tumor recurrence (40–43). Even after several years of research, the origin of these cells still remains controversial. Our data shows that Rb-TS inactivation leads to transition of adult astrocytes into plastic, proliferative, multipotent, self-renewable cells providing a potential alternative process for the origin for brain TICs and eliminates the involvement of adult stem cells in our GBM model.

TCGA data shows that about 88% of GBM cases show RTK/RAS/PI3K pathway altered and about 10% of cases with NF1 mutations. Given the importance of this pathway, our TR GBM model layers Kras activation over Rb-TS inactivation (9). When we probed the mice with just Kras activation for progenitor-like transition, we failed to observe any changes of differentiation status in the cortical cells. However, combination with Rb-TS inactivation showed identical change of progenitor-like transition as observed in Rb-TS inactivated alone. Another study (44) showed similar results, where H-Ras V12 alone failed to initiate dedifferentiation but showed a cooperative effect with p53 deficiency to initiate the dedifferentiation in astrocytes and neurons. In our system, Rb-TS inactivation alone was necessary and sufficient to eliminate the G1-S arrest and initiate progenitor-like transition. Further investigation is required to precisely identify the partners involved downstream of Rb-TS inactivation to bring about this transition.

**Figure 6:**
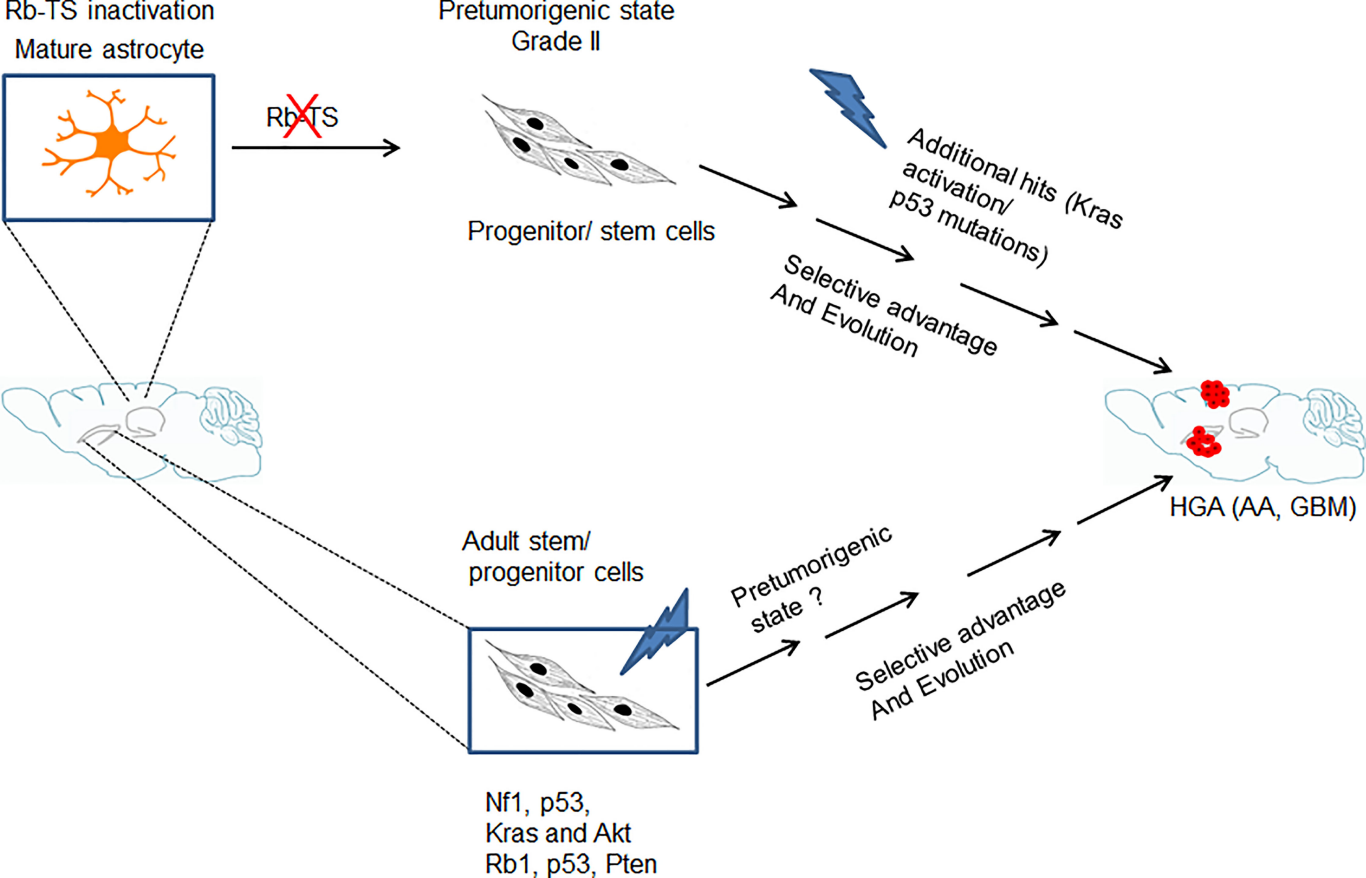
Model showing genetic alteration and cell type specific pathways for GBM development. Transition of mature astrocytes by Rb-TS inactivated and generation of pretumorigenic state that after accumulation of additional spontaneous mutations, along with selective pressure and evolution initiates HGA.

The abnormal proliferation initiated by Rb-TS inactivation further acted as a source for stochastic mutations which upon selection, transform the pretumorigenic progenitor-like cells into tumorigenic cells developing into HGAs (**Figure 6**). The uniqueness of this GBM model is the development of GBM from a single genetic alteration of Rb-TS inactivation. A situation likely similar to humans, where a single or a few genetic alterations gradually evolve to develop HGA. We are in the process of understanding the spontaneous mutations which drive these progenitor-like cells to form HGAs.

### Relevance of Our Work

In addition to the well-studied example of retinoblastoma caused by Rb-TS inhibition alone, Rb loss is also known to initiate small cell lung cancer (SCLC) in 90% of the human cases. There are several mouse cancer models signifying the role of Rb in tumorigenesis in combination with other genetic alterations. In brain cancers, using the developmental mouse model and GFAP Cre driver, induced loss of Rb and p53 has been shown to initiate medulloblastoma (45). TCGA for GBM shows that the Rb pathway is disrupted in 78% of the GBM cases (46). Other than the homozygous deletion which is present in 11% of cases, all the other alterations are upstream of Rb family which in the mouse system, will lead to complete disruption of the three family members, a situation mimicked in our TR HGA GEMM (46). Thus, transition of mature astrocytes to a more plastic-progenitor stage is a plausible scenario with subsequent hits resulting in the transformed TICs capable of initiating HGAs. Interestingly, the GBM mouse model described by Brandner’s group (7) utilizes genetic alterations of p53, Rb, and PTEN to initiate the disease and suggests adult stem cells as the cells of origin. Rb loss alone failed to initiate a phenotype in Baker’s mouse model (47). These observed differences in phenotypes could be contributed by the complimentary roles of p107 and p130.

In our systemically induced HGA model, adult stem cells in the brain were also genetically altered for Rb-TS inactivation but did not show any selective advantage over mature astrocytes in inducing proliferation. Whereas, several studies, including a recent study (48) using alterartions in Pten, Egfr and p53 have demostrated that adult stem cells to be the cell of origin. One possible explanation for this difference could be the genetic alteration used in our model, suggesting that different genetic alterations could target different cells in the brain. Our conclusion does not exclude the possibility of HGA initiation in other situations from adult stem cells as these are already existing stem cells in the brain with the required flexibility and proliferative potential which upon transformation could generate TICs. Our study proposes a novel pathway of transitioning adult astrocytes, suggesting that we need to broaden our approach in treating this disease. It also suggests mature astrocytes as new targets for treatment and a possibility of altering the tumor microenvironment to force differentiation of TICs as an attractive treatment option. Disruption of Rb network is a common event in several cancers and hence, our study also provides a potential generalized pathway for cancer initiation in multiple cancers.

## Data Availability Statement

The datasets presented in this study can be found in online repositories. The names of the repository/repositories and accession number(s) can be found below: https://www-ncbi-nlm-nih-gov.ezproxy.u-pec.fr/geo/query/acc.cgi?acc=GSE26069, GEO26069.

## Ethics Statement

The animal study was reviewed and approved by Institutional Animal Care and Use Committees at the National Cancer Institute at Frederick, MD.

## Author Contributions

ASA designed, performed and analyzed the experiments, TS performed the experiments. RB performed the microarray and GSEA analysis, LL and NO’S performed animal experiments, YS developed the model and analyzed the data, TVD generated the hypothesis, design the experiments and analyzed the data, ASA and TVD wrote the manuscript. All authors contributed to the article and approved the submitted version.

## Funding

This research was supported by the Intramural Research Program of the NCI-Center for Cancer Research.

## Conflict of Interest

The authors declare that the research was conducted in the absence of any commercial or financial relationships that could be construed as a potential conflict of interest.

## Publisher’s Note

All claims expressed in this article are solely those of the authors and do not necessarily represent those of their affiliated organizations, or those of the publisher, the editors and the reviewers. Any product that may be evaluated in this article, or claim that may be made by its manufacturer, is not guaranteed or endorsed by the publisher.
