# Supplementary material for: Abrogation of Rb Tumor Suppression Initiates GBM in Differentiated Astrocytes by Driving a Progenitor Cell Program
Source: Front Oncol. 2022 Jun 24;12:904479. doi: 10.3389/fonc.2022.904479 (PMC9263358; doi:10.3389/fonc.2022.904479)
Supplement: Supplementary file 1 [file DataSheet_1.docx]

**Supplementary Material and Methods and Figure legends**

**Supplementary Material and Methods**

**Neurosphere culturing**

Cortex or SVZ was obtained from appropriately aged adult mice as described elsewhere ([32](#_ENREF_32)). Neurosphere culturing was performed as described previously ([49](#_ENREF_51)). Briefly, tissue was washed three times in DPBS and incubated with enzyme mix (1.33mg/ml Trypsin, 0.66mg/ml Type 1-S Hyaluronidase and 0.13mg/ml Kynurenic Acid, Sigma Aldrich) for 10 minutes at 37^o^C, with intermittent triturating. After incubating with Trypsin inhibitor (Sigma Aldrich) for additional 10min at 37^o^C cells were cultured at appropriate dilutions in neurosphere growth media containing 20ng/ml murine EGF (BD Biosciences) and recombinant human 20ng/ml FGF basic (Pepro Tech) on ultra-low attachment plates or dishes (Corning Incorporated). Every alternate day media was replaced with fresh media containing murine EGF and recombinant human FGF. Cultures were subcultured with dissociation solution (Sigma Aldrich) after 14 days or when spheres attained ~50-150um in size. For neurosphere clonal assay, single cell suspension of cortex or SVZ was cultured in 96 wells at 8cells/ well dilution for 4 generations. For regular culturing, cells were seeded at 1x10^4^ to 3x10^4^ cells/cm^2^ (5[0](#_ENREF_52)). Neurosphere cells used in assays were under 30 passages.

**Inducing in vitro differentiation**

Prior to culturing the cells, chamber slides (Thomas Scientific) were coated with 50ug/ml of Laminin (Sigma Aldrich) for 1 hour in a 37^o^C/5%CO_2_ incubator. Neurospheres were dissociated into a single cell suspension and cultured in 2D in neurosphere growth media. For differentiation, the media was changed to neurosphere growth media without growth factors and with the addition of 1% fetal Bovine serum (Atlanta Biologicals) for 10 days.

**Immunohistochemistry**

Brain tissues were fixed in 10%NBF, paraffin blocks were sectioned at 6um thickness for Haematoxylin and Eosin or immunohistochemistry. Sections were de-paraffinized, EtOH dehydrated and antigen unmasked by 1mM citric acid treatment. For all antibodies except SV40 T antigen, sections were blocked with 10% donkey serum (Jackson Immunochemicals) or 10% goat serum (Vector Laboratories) in PBS for 1 hour. Primary antibodies were diluted accordingly in 0.1% BSA in PBS with 0.001%Tween 20 (Sigma Aldrich) (PBST) for 1 hour. Secondary antibodies were diluted in PBST to appropriate dilutions and incubated for 30 minutes. For SV40 T antigen, sections were blocked in 1.5% horse serum (Vector Laboratories) in PBS for 1 hour. Biotinylated anti-mouse secondary antibody (Vector laboratories) was diluted to appropriate concentration in blocking buffer, incubated for 30 minutes. All the antibodies used are in the antibodies list in Supplementary Materials.

**Immunocytochemistry**

Cells were fixed in 4% paraformaldehyde for 15 minutes and permeabilized with 0.2% Triton X-100 for 5 minutes. Cells were blocked with 10% donkey serum or 10% goat serum in PBS for 1 hour. The primary antibodies were diluted in appropriate concentrations in 5% blocking serum /0.3%BSA/PBST (T- Tween 20, 0.1%) and incubated for 1 hour. The secondary antibodies were diluted in PBST and incubated for 30minutes. Washes were performed in PBST. All immunocytochemistry was performed at room temperature.

**In vitro astrocytes culturing**

Cortex tissue from 5 to 8 day old pups was mechanically disrupted into single cell suspension by trituration and re-suspended in AM-a media (ScienCell Research Laboratories) with 3.5% total FBS for 24 hrs and later reduced to 2 % FBS. For mature astrocytes, cells were cultured until astrocytes stopped dividing (approximately 2-4 passages) and then profiled for mature astrocyte markers.

**In vitro dedifferentiation of astrocytes**

Mature astrocytes were cultured in AM-a media and monitored daily for cell division. Upon confirming the complete termination of cell division, astrocytes were either treated with 0.2uM 4-Hydroxy Tamoxifen (4OHT) or the vehicle twice in 72 hrs. After 7 days of incubation, cells were either fixed for immunostaining or utilized for neurosphere assay.

**Astrocyte reprogramming with lentivirus-cre**

T or TR astrocytes used for reprogramming were treated with lentivirus expressing Cre recombinase for 48 hours in AM-a media. After infection, the astrocytes were incubated for different time points in AM-a media, and then used for cloning or immunocytochemistry.

**qPCR:** RNA was isolated from neurosphere cell lines and cortical tissues acquired at different time points by RNeasy kit (Qiagen). 1ug of RNA was converted to cDNA and qPCR was run using either iTaq Fast SYBR Green Super mix with ROX (Biorad) or by Taqman probes (Applied Biosystems) in 7500 Fast Real Time (Applied Biosystems). List of primers and probes is given in Supplementary Materials. Beta-actin was used as an internal control.

**Sample preparation and microarray data generation**

Tissue samples were stored in RNA later (LifeTech, CA) until RNA isolation. Total RNA was extracted using Trizol (LifeTech, CA) for each tissue sample and the quality of RNA was estimated on the Bioanalyzer. The RNA was labeled with one-color Agilent kits according to manufacturer’s protocols. The labeled RNA was hybridized to custom designed Agilent arrays (GPL11318) that contained a total number of 60893 probes for 24510 unique Entrez Gene ids. In order to avoid any batch effects, we processed all the samples on the same day, on two arrays. The hybridized arrays were scanned on Agilent scanner and these fluorescence images were then processed with Agilent Feature Extraction software.

**Supplementary Material**

**Antibody list:**

**Antibody** **Company** **Catalog number/clone**

SV40 T Antigen(Ab-2)(mouse) Calbiochem DP02 clone PAb416

KI-67 (rabbit) Abcam ab15580

Nestin (Chicken) Novus NB100-1604

Nestin (rat) Abcam ab81462

GFAP (rabbit) DAKO Z0334

S100β (rabbit) Abcam ab52642

CXCR4 (rabbit) Abcam ab2074

GS (rabbit) Abcam ab49873

GalC (rabbit) Millipore AB142

NG2 (rabbit) Millipore AB5320

Beta tubulin class III (rabbit) Millipore 04-1049

MAP2 Millipore AB5622

**Primer List for qPCR:**

Gli2-F 5’ GAG CCA CCC CAG CGT AGA CA-3’

Gli2-R 5’GCC CCA GGT CGC ACT CTA G-3’

Notch1-F 5’ AGT GTG ACC CAG ACC TTG TGA-3’

Notch1-R 5’AGT GGC TGG AAA GGG ACT TG-3’

Notch2-F 5’ CCC AAG GAC TGC GAG TCA GG-3’

Notch2-R 5’ GGC AGC GGC AGG AAT AGT GA-3’

mBactin-F 5’ CGA GGC CCA GAG CAA GAG-3’

mBactin-R 5’ CGT CCC AGT TGG TAA CAA TGC-3’

Ezh2-Taqman probe- Mm00468464_m1

Bcl2l12-Taqman probe-Mm00511671_m1

**References**

1. Pacey L, Stead S, Gleave J, Tomczyk K, Doering L. Neural Stem Cell Culture: Neurosphere Generation, Microscopical Analysis and Cryopreservation. protocolexchange (2006). doi: <https://doi.org/10.1038/nprot.2006.215>
2. Pellegatta S, Poliani PL, Corno D, Menghi F, Ghielmetti F, Suarez-Merino B, et al. Neurospheres Enriched in Cancer Stem-Like Cells are Highly Effective in Eliciting a Dendritic Cell-Mediated Immune Response Against Malignant Gliomas. Cancer Res (2006) 66:10247–52. doi: 10.1158/0008-5472.CAN-06- 2048
