# Supplementary figures and images for: Abrogation of Rb Tumor Suppression Initiates GBM in Differentiated Astrocytes by Driving a Progenitor Cell Program
Source: Front Oncol. 2022 Jun 24;12:904479. doi: 10.3389/fonc.2022.904479 (PMC9263358; doi:10.3389/fonc.2022.904479)

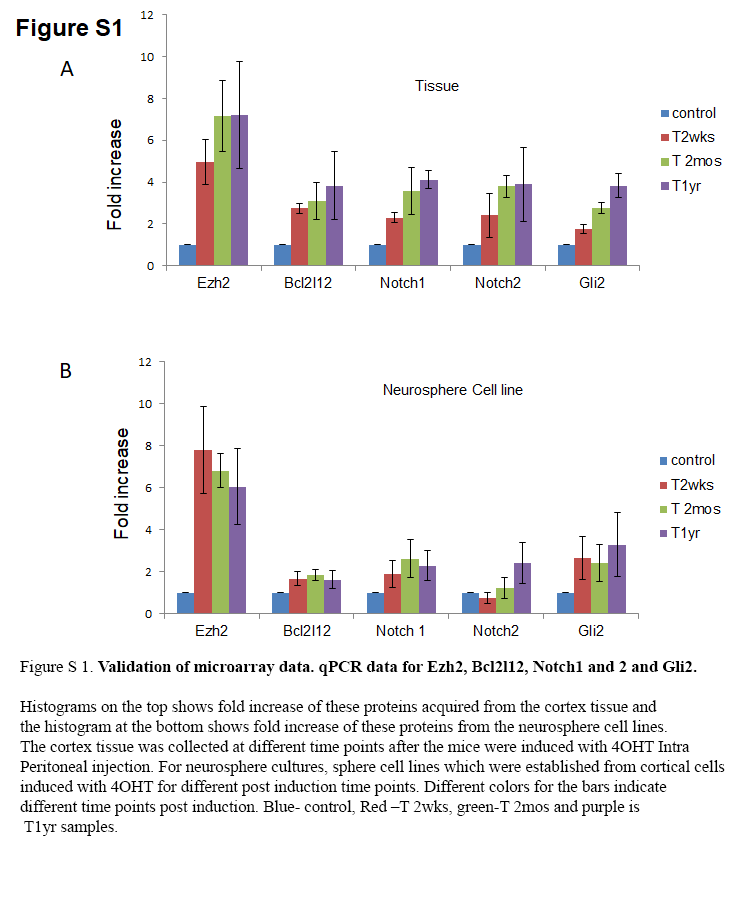

Supplement: Supplementary file 2 [file Image_1.tif]

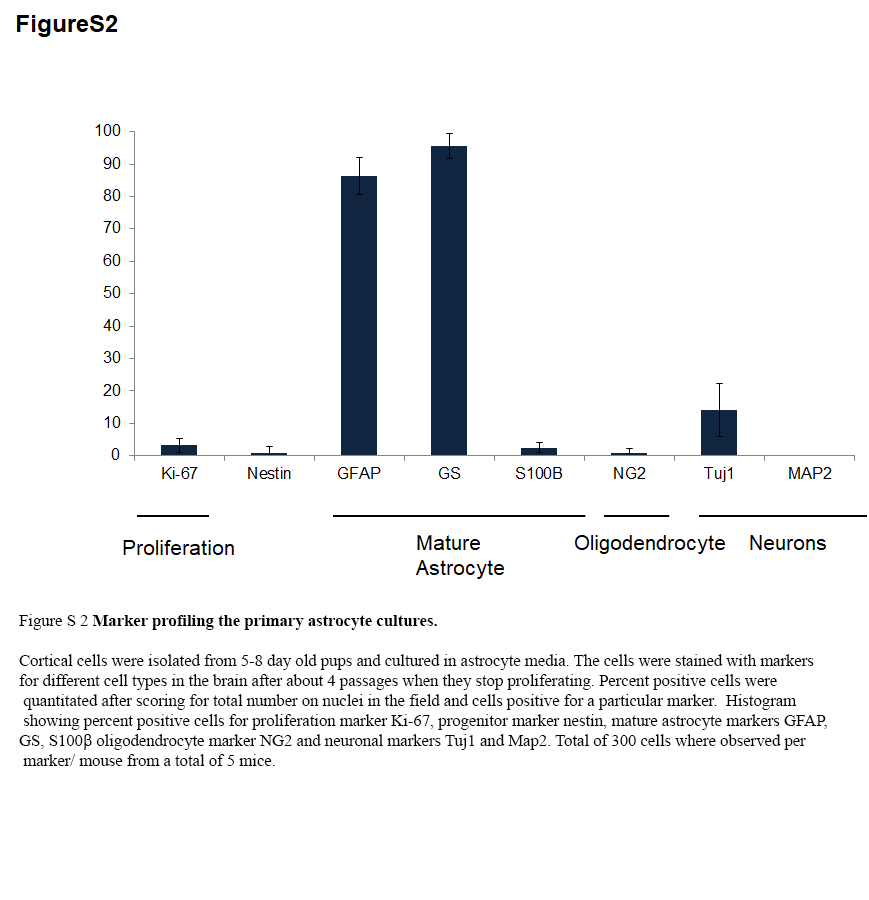

Supplement: Supplementary file 3 [file Image_2.tif]

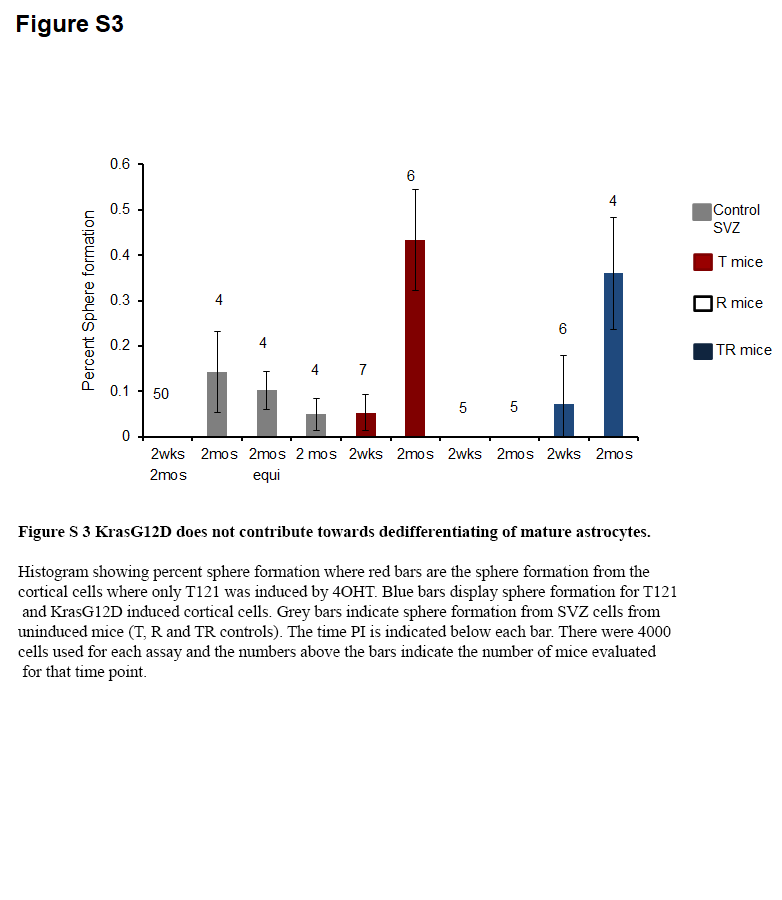

Supplement: Supplementary file 4 [file Image_3.tif]
